# A Serious Charge

**Published:** 1900-04

**Authors:** 


### EDITORIALS.

### A SERIOUS CHARGE.

On another page of this issue will be found a statement of the arrest of one of Philadelphia's milk inspectors on a very serious charge, that of extorting money from a milk producer to suppress legal proceedings for the alleged violation. The field of all inspections is at all times one of great responsibility and temptation. The use of bribes, gifts, the granting of favors and many perquisites are ever at the taking or command of those in official place, and one must be strong, indeed, not to in some way find himself obligated to forget duty at times or to play the lenient rôle toward some violator of the law. It is the first charge openly made, and the record of this official has been a clear one, and judgment as to his guilt should be suspended until a trial by jury. The desire at times to trap earnest, vigilant officials by unscrupulous dealers is well known, and we are hopeful that this may prove true in this case.

At all events, we sincerely trust that this case will be pushed to prompt determination, and whoever may be the guilty parties be meted out the severe punishment such actions deserve.

In these days of bribery and extortion in high and low public places, it makes one shudder at times at the thought of the failure of popular government, national, State, and municipal. Punishment of a severe character is too rarely meted out, and the freedom given to those who violate the oath of public office is a danger and a menace to successful government that must soon be stamped out.

